# Role of Probiotics in the Management of COVID-19: A Computational Perspective

**DOI:** 10.3390/nu14020274

**Published:** 2022-01-10

**Authors:** Quang Vo Nguyen, Li Chuin Chong, Yan-Yan Hor, Lee-Ching Lew, Irfan A. Rather, Sy-Bing Choi

**Affiliations:** 1 Centre for Bioinformatics, School of Data Sciences, Perdana University, Suite 9.2, 9th Floor, Wisma Chase Perdana, Changkat Semantan, Wilayah Persekutuan, Kuala Lumpur 50490, Malaysia; 21210002@perdanauniversity.edu.my; 2 Beykoz Institute of Life Sciences and Biotechnology, Bezmialem Vakif University, Beykoz, Istanbul 34820, Turkey; lichuinchong@gmail.com; 3 Department of Biotechnology, Yeungnam University, 280 Daehak-Ro, Gyeongsan 38541, Gyeongbuk, Korea; yanyanhor@yu.ac.kr; 4 Probionic Corporation, Jeonbuk Institute for Food-Bioindustry, Jeonju 54810, Korea; lewleeching@probionic.com; 5 Department of Biological Sciences, Faculty of Science, King Abdulaziz University, P.O. Box 80203, Jeddah 21589, Saudi Arabia; 6 Center of Excellence in Bionanoscience Research, King Abdulaziz University, P.O. Box 80203, Jeddah 21589, Saudi Arabia

**Keywords:** probiotics, SARS-CoV-2, COVID-19, gut-lung axis, microbiome, computational approach, molecular docking

## Abstract

Coronavirus disease 2019 (COVID-19) was declared a pandemic at the beginning of 2020, causing millions of deaths worldwide. Millions of vaccine doses have been administered worldwide; however, outbreaks continue. Probiotics are known to restore a stable gut microbiota by regulating innate and adaptive immunity within the gut, demonstrating the possibility that they may be used to combat COVID-19 because of several pieces of evidence suggesting that COVID-19 has an adverse impact on gut microbiota dysbiosis. Thus, probiotics and their metabolites with known antiviral properties may be used as an adjunctive treatment to combat COVID-19. Several clinical trials have revealed the efficacy of probiotics and their metabolites in treating patients with SARS-CoV-2. However, its molecular mechanism has not been unraveled. The availability of abundant data resources and computational methods has significantly changed research finding molecular insights between probiotics and COVID-19. This review highlights computational approaches involving microbiome-based approaches and ensemble-driven docking approaches, as well as a case study proving the effects of probiotic metabolites on SARS-CoV-2.

## 1. Introduction

The ongoing onslaught of the COVID-19 pandemic has threatened human life and health with a catastrophic impact on global financial and medical burdens [1]. There were nearly 260 million cumulative confirmed COVID-19 cases, with over five million deaths (https://www.who.int/emergencies/diseases/novel-coronavirus-2019, accessed on 28 November 2021). Although vaccines are available against severe acute respiratory syndrome coronavirus 2 (SARS-CoV-2)—the causative agent of COVID-19, the number of cases is still growing worldwide, especially when control measures are relaxed [2]. Recently (as of 28 November 2021—time of analysis), a deadly super variant of SARS-CoV-2 with around 50 mutations overall, called Omicron (B.1.1.529 lineage), has been classified as the “Variant of Concern” (VOC) by World Health Organization (WHO) [3]. The VOC is the WHO’s top category of worrying COVID-19 variant. Thus, there is an urgent need to develop complementary strategies to derive preventive and supportive roles.

COVID-19 has a broad spectrum of clinical manifestations, ranging from asymptomatic, mild symptoms (cough and fever, among others) to severe illness that may lead to death [4]. Emerging evidence shows that many infections of COVID-19 are asymptomatic—there has been positive detection of the nucleic acid of SARS-CoV-2 by use of reverse transcriptase-polymerase chain reaction (RT-PCR) in samples of patients with no clinical symptoms [5]. Such patients can serve as a source of disease dissemination by transmitting the virus to others, leading to the continued spread of COVID-19. Common presenting symptoms of COVID-19 include fever, tiredness, dry cough, rhinorrhea, gastrointestinal (GI) symptoms (e.g., diarrhea and nausea), and dyspnea with hypoxemia [6]. Some patients presented with severe clinical signs, such as pneumonia, lung edema, multi-organ malfunction, and acute respiratory disease syndrome (ARDS). As such, COVID-19 may require a multidimensional therapeutic approach, transitioning from virus-targeted approaches (early stage of infection) to immunomodulation (late-onset stage) [7].

It has been shown that COVID-19 patients demonstrate intestinal microbial dysbiosis, even six months after recovery [8]. A malnutrition ecosystem of COVID-19 patients with decreased probiotics, such as *Lactobacillus* and *Bifidobacterium*, may hinder recovery [9]. This suggests that the fine-tuning of host-microbiota balance in the lung and gut could be useful in fighting against COVID-19. Given the ability of probiotics in immunomodulation [10], anti-inflammatory [11], antioxidant [12], and antiviral [13] effects, the use of probiotics may be a way to support the reconstitution of the gut microbiota. These benefits could be elucidated if the molecular insight is known on how probiotics or their metabolites can prevent or reduce the SARS-CoV-2 infection.

To date, the computational approach has been immensely developing and becoming an integral part of disease-related projects, along with the development of computational technology, algorithms, and tools. Amid the COVID-19 pandemic, various movement control measures are implemented to break the chain of COVID-19 infection. This situation had halted most of the conventional laboratories’ research. The computational methods, such as computer-aided drug discovery and utilizing machine learning for the predictive models, have been proven effective in researching the impacts of probiotics on SARS-CoV-2, from the available wide range of data to ample aspects pertaining to microbiome and protein structure. Herein, we review the potential role of probiotics in the management of COVID-19 and focus on computational approaches that may become indispensable in providing significant understanding in probiotics research.

## 2. Probiotics against Viral Infection

Probiotics are a beneficial live microorganism which, when administrated in sufficient quantity (at least 106 viable CFU/g), are known to participate in metabolism, improving the microbial balance in the gut [14,15,16]. Probiotics of mainly the strains of lactic acid bacteria, in particular *Lactobacillus* and *Bifidobacterium* genera, show various health effects [17]. Their well-established properties have been extensively studied, primarily modulating the gut microbiota via the growth suppression of opportunistic bacteria [18]. Beyond the gut, probiotics have been reported to exert beneficial health effects through several potential mechanisms, including immunomodulation, epithelial barrier function maintenance, and signal transduction modulation [19].

**Figure 1:**
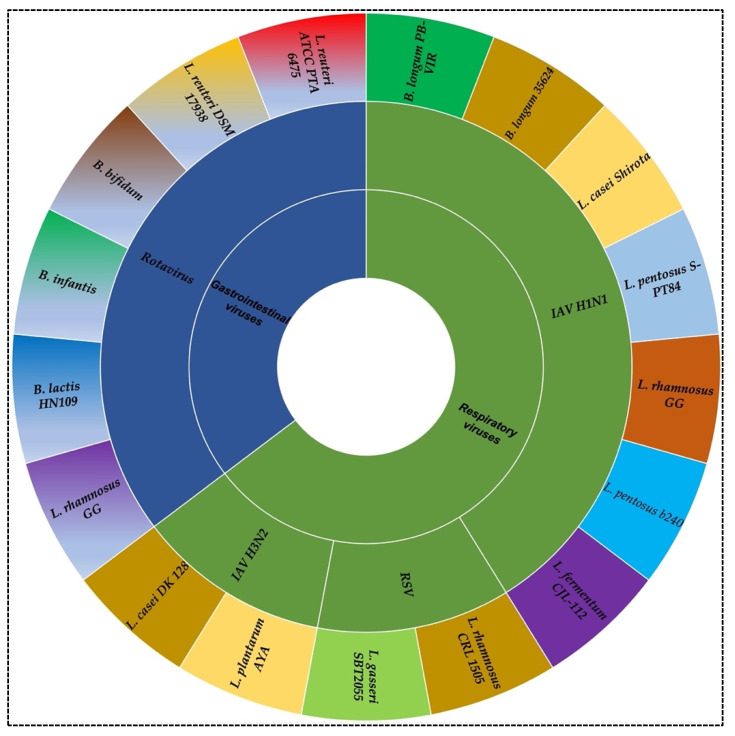
Probiotics strains against respiratory (influenza A virus H1N1, H3N2, and respiratory syncytial virus) and gastrointestinal viruses (rotavirus). The figure represents some examples of different strains of *Lactobacillus* and *Bifidobacterium* studied for the antiviral effects against viruses.

Viral infectious diseases are a primary contributor to the global burden of death and disability [20]. Both developed and developing countries struggle against the alarming rise in infectious diseases [21]. The best current example of this global threat is the novel COVID-19, with millions of people afflicted. Despite the success of therapeutic and preventive strategies against the disease, concerns remain with the continued reporting of new viral variants [22,23]. As a result of infectious disease, profound damage to multiple organs, including the respiratory tract, liver, colon, and more, supports the urgent need for alternative strategies against viral infection. Notably, a diverse microbial community inhabits about every part of the human body, mainly in the intestine [24]. A stable and healthy microbial community is able to protect the human host from a variety of pathogen infections by preventing viral infectivity through a diverse mechanism and exerting substantial inhibitory effects [25]. As such, probiotics serve as a complementary strategy, given their beneficial effects against viral disease by potentiating immune response, maintaining the epithelial barrier, and binding to the pathogen to skew its attachment. These antiviral effects of different strains of *Lactobacillus* and *Bifidobacterium* have been studied on both gastrointestinal and respiratory viruses (Figure 1).

More than 70% of the body’s immune cells are located in the GI tract, indicating a direct connection between the immune system and intestinal microflora, which provide some relationships with GI viruses [26]. Some studies have revealed the immunomodulation effect of *Lactobacillus* and *Bifidobacterium* against rotavirus (RV), one of the leading global causes of life-threatening diarrhea in children under the age of five [27,28]. RV alters the human gut microbiome by shifting the dominant phylum from *Bacteroidetes* to *Firmicutes*, decreasing bacterial diversity, and increasing opportunistic pathogens, such as the genera *Shigella* [29]. Both *Lactobacillus reuteri* strains ATCC PTA 6475 and DSM 17938 augmented mucosal RV-specific antibodies in infected neonatal mice and attenuated diarrhea symptoms [30]. The nutritional status of body mass index—normal weight, underweight, and overweight—may impact the response of probiotics on RV. Underweight mice had fewer RV-specific antibodies than the other two groups. In another study, the combination of *Lactobacillus rhamnosus* GG with specific bovine colostrum-derived immunoglobulins significantly decreased the severity and duration of diarrhea in an infant mouse model [31]. Both *Bifidobacterium bifidum* and *Bifidobacterium infantis* contributed to delaying clinical diarrhea in RV-infected mice [32].

Furthermore, the probiotics also enhanced the immune response, resulting in a high elevation of specific IgA. *Bifidobacterium longum* subspecies *infantis*, which was incubated in cell cultures prior to infection, showed the ability to reduce RV infectivity in both HT-29 and MA-104 cells in vitro [33]. Additionally, the in vivo study applied on a BALB/c mouse model revealed that viral shedding in stools was decreased in probiotic-fed mice challenged with RV, compared to control mice. In experiments with piglet models, *Bifidobacterium lactis* HN019 diminished the severity of diarrhea, and fecal RV concentration was also reduced [34]. Additionally, *Bifidobacterium lactis* HN019 elevated the immune response in infected piglets; particularly, specific IgG, IgA, and IgM concentrations in fecal samples were observed. *Lactobacillus rhamnosus* GG also decreased the severity of RV infection in a gnotobiotic pig model [35]. According to previous studies, despite the success of vaccine development against RV, the gut microbiota for unvaccinated and vaccinated people has no differences [36,37]. The combination of probiotics and vaccination has been studied, proving that able to improve the gut microbiota efficiently. *Lactobacillus acidophilus* increases the immunogenicity of an oral RV vaccine, enhancing the IgG and IgA antibody-secreting cell responses [38]. *Lactobacillus rhamnosus* GG and *Bifidobacterium lactis* Bb12 act as immunostimulants for RV vaccine via differential toll-like receptor signaling which modulated dendritic cell responses [39]. Therefore, probiotics are still considered a need as an adjuvant for the RV vaccine.

Besides GI viruses, probiotics and microbiota have been proven to have an antiviral effect on respiratory viruses, including the fatal seasonal scourge—influenza virus [40]. Influenza causes about 20,000 deaths based on the Centers for Disease Control and Prevention (CDC) estimation for 2019–2020 (https://www.cdc.gov/flu/about/burden/index.html, accessed on 28 November 2021). A mounting body of evidence shows that nasally and orally administered probiotics can enhance the resistance against respiratory viral infections. A heat-killed *Lactobacillus casei* DK128 showed an effect on mice infected with H3N2, resulting in a lower viral titer in heat-killed DK128 treated mice compared to the mock-treated mice group [41]. Moreover, a higher quantity of alveolar macrophage cells was found in the lung and airways of heat-killed DK128 treated mice. Alveolar macrophages, in the interphase between lung tissues and air, can provide the first line of innate immunity against the influenza virus [42]. In another study, alveolar macrophages were shown to release many inflammatory cytokines that helped control viral replication [43]. The *Lactobacillus rhamnosus* GG administration was carried out to investigate the anti-H1N1 ability [44]. The survival rate was roughly 60% and 20% for *Lactobacillus rhamnosus* GG-treated mice and the control groups, respectively. Of note, a significant increase in NK cell activity was observed in *Lactobacillus rhamnosus* GG group compared to untreated group. Another strain of probiotics, *Lactobacillus pentosus* S-PT84, also exerted a strong induction of IL-12 and high IFN-γ production in mediastinal lymph node cells, contributing to the high improvement of survival rates and reducing H1N1 virus titer in bronchoalveolar lavage fluid [45]. Similarly, the intranasal administration of *Lactobacillus casei* strain Shirota stimulated the IL-12 production and NK cell activity, resulting in adequate protection against H1N1 infection [46]. Additionally, there was an increase of IgA in the nasally administered mice with *Lactobacillus fermentum* CJL-112, leading to the antiviral effect against influenza A/NWS/33 (H1N1) [47]. In another study, *Lactobacillus plantarum* AYA induced the increase of IgA production in lung and small intestine in the H3N2 infected mice [48]. The rise of IgA and IgG production was also observed in the H1N1-infected mice treated with *Lactobacillus pentosus* strain b240 [49]. Two other strains of *Bifidobacterium*, including *Bifidobacterium longum* 35624 and *Bifidobacterium longum* PB-VIR, also induced the reduction of viral load and improved survival rate in mice challenged with influenza virus strain PR8 (A/Puerto Rico8/34, H1N1) [50].

Besides the influenza virus, the human respiratory syncytial virus (RSV) also showed a specific correlation with the gut microbiota and probiotics [51,52]. RSV is an enveloped negative-strand, non-segmented RNA virus of the *Paramyxoviridae* family, which primarily causes severe respiratory disease in infants and children [53]. RSV-related lower respiratory tract infection was associated with roughly 48,000–74,500 deaths in children aged less than 5 years in 2015 [54]. The oral administration of *Lactobacillus gasseri* SBT2055 (LG2055) leads to a significant decrease in RSV titer in the lung [55]. Another probiotic strain, *Lactobacillus rhamnosus* CRL1505, also showed the capacity of reducing lung viral in a study where CRL1505 was orally administered to infant mice infected with RSV [56]. It was found that the increase in IL-10 and IFN-γ secretion after CRL1505 treatment would modulate the pulmonary innate immunity, leading to the activation of dendritic cells and the generation of Th1 cells. Similar results were also obtained when CRL1505 was nasally administered [57].

## 3. Gut-Lung Axis Associated with COVID-19

**Figure 2:**
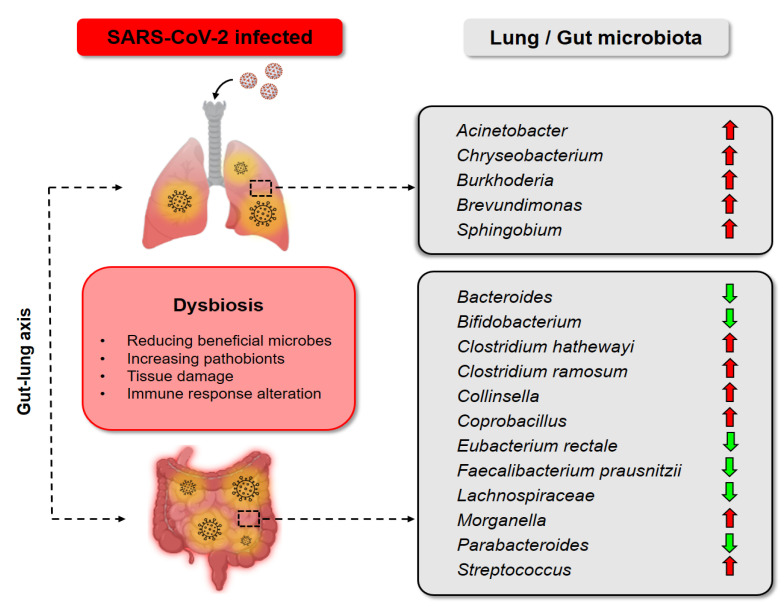
Dysbiosis of gut and lung in COVID-19 patients. In the lung of SARS-CoV-2 infected patients, *Acinetobacter*, *Chryseobacterium*, *Burkhoderia*, *Brevudimonas*, and *Sphingobium* were prevalent [74]. The gut microbiota of COVID-19 patients was also altered, with the decrease of *Bacteroides* [69], *Bifidobacterium* [68], *Eubacterium rectale* [68], *Faecalibacterium prausnitzii* [73], *Lachnospiraceae* [69], *Parabacteroides* [69], and the increase of *Clostridium hathewayi* [71], *Clostridium ramosum* [71], *Collinsella* [69], *Coprobacillus* [71], *Morganella* [69], *Streptococcus* [69].

SARS-CoV-2 primarily infects the respiratory tract by attaching to the angiotensin-converting enzyme 2 (ACE2) receptor [58]. This receptor is expressed in different organs and is highly expressed on the surface of the type-II alveolar epithelial cells and airway epithelial cells. Despite the respiratory system being the leading target site of the virus, the GI tract is also an important target, contributing to GI symptoms, including nausea, diarrhea, and vomiting [59]. Studies have shown that coronavirus viral RNA can be detected in urine and fecal samples from COVID-19 patients [60]. The alteration of intestinal flora composition (dysbiosis) has been reported for COVID-19 patients (Figure 2). These findings suggest the importance of understanding the gut-lung axis (GLA) for the management of COVID-19.

The GLA, herein, refers to bidirectional interactions between the respiratory mucosa and the gut microbiota, with the ultimate goal of modulating the immune response [61]. It is widely known that the gut has a large amount of microbiota exerting a marked effect on host homeostasis and disease [62]. A healthy lung has also been demonstrated to have its own specific microbiota, including *Prevotella*, *Streptococcus*, *Veillonella*, *Fusobacterium,* and *Haemophilus* [63,64]. Although there is a limited understanding of the impact on the microbiome in the disease etiology, gut dysbiosis has been proved to increase the risk of diseases [65]. For instance, inflammatory bowel disease (IBD) causes inflammation and promotes respiratory tract infection, supporting the crosstalk between lung and gut microbiota [66]. A high percentage of COVID-19 patients demonstrate GI symptoms. The gut bacterial diversity of COVID-19 patients was significantly reduced compared with healthy controls [67]. Several gut commensals with immunomodulatory effects, such as *Eubacterium rectale* and *Bifidobacterium*, were underrepresented in patients [68], while *Collinsella*, *Streptococcus*, and *Morganella* were significantly increased in patients with high SARS-CoV-2 infectivity [69]. Zuo and colleagues found increased levels of *Parabacteroides*, *Bacteroides*, and *Lachnospiraceae* in patients with low or no SARS-CoV-2 infectivity. These bacteria are able to produce short-chain fatty acids, which play an important role in boosting the host immunity [70]. The relative abundance of *Coprobacillus*, *Clostridium ramosum*, and *Clostridium hathewayi* was positively correlated to COVID-19 severity. Conversely, *Faecalibacterium prausnitzii* and *Bacteroides* were inversely correlated to COVID-19 severity [71,72,73]. Strikingly, the microbiota composition in community-acquired pneumonia (CAP) patients is similar to COVID-19 patients.

### 3.1. Rationale of Probiotics as an Adjunctive Treatment for COVID-19

Gut microbiota abnormality increases the susceptibility of an individual to various diseases. Emerging evidence suggests that probiotics are beneficial in the control of COVID-19. Probiotics are known for restoring stable gut microbiota through the interaction and coordination of the intestinal innate and adaptive immunity. Various types of cells, such as mast cells, natural killer (NK) cells, and macrophages, interact with the gut microbiome to regulate innate immunity. For instance, antigen-presenting cells, comprising dendritic cells in the Peyer’s patches of the intestine, Langerhans cells, and macrophages, have some tolerant immunogenic properties [75]. B and T lymphocytes are mainly involved in the adaptive system. The increased level of naïve T helper (Th) lymphocytes and decreased levels of NK cells, B lymphocytes, and memory Th lymphocytes have been observed in COVID-19 patients. The immune homeostasis of the gut can affect the immunity of the lung via GLA. This is probably through a deregulated immune response, with an increase of IFN-γ, IL-6, CCL2, and a decrease of regulatory T cells in the lung and GI tract [76].

Probiotics are considered harmless, originating from the fermentation of food—an ancient form of food preservation and widely used as food additives [77,78]. Probiotics are measured in colony-forming units (CFU), indicating the number of viable cells [79]. The amount of probiotics is usually written as 5 × 10^9^ for 5 billion CFU or 1 × 10^10^ for 10 billion CFU on the commercially available probiotics products. Various probiotic products contain different probiotic amounts, but the standard effective dosages for adults are from 10 to 20 billion CFU. In comparison, the dosages for children are recommended at around 5 to 10 billion CFU [80]. The higher the dosages of probiotics used, the higher the beneficial outcome may be expected. There is no evidence that overdosage of probiotics is of health risk concern [80].

Several concerns of probiotics safety should be considered, such as antibiotic resistance and their toxicity on the GI tract [81]. Although probiotics have been shown not to exhibit toxic effects, there are still rare cases of bacteremia involving probiotics, observed in immunocompromised individuals [77]. The guidelines for the safety assessment of probiotics are highly stringent, particularly in relation to the identification of the risk factors for both probiotics and the host, followed by the verification of the risks in the interaction between the used probiotics and the host [82]. Hence, this does not only evaluate the beneficial effects of probiotics but also their side effects. Moreover, to further evaluate the safety of probiotic products, epidemiological surveillance of adverse incidents in human use is implemented [83]. Accordingly, the usage of probiotics as an adjunctive treatment against COVID-19 can be expected in the future as a modulator of immune response to decrease pathogenic microbiome in the host.

### 3.2. Clinical Evidence That Supports Probiotics as a Promising Anti-COVID-19 Strategy

The consumption of probiotics is considered to relieve COVID-19 symptoms by boosting immune host response and improving gut microbiota [84]. The use of probiotics may indicate its ability to combat SARS-CoV-2 or its associated symptoms through evaluation of antiviral and anti-inflammatory probiotic effects, in vitro, in vivo, and clinically [85]. A review suggested that enhancing the intestinal microbiota profile by personalized diet and supplementation, especially with probiotics, can improve the immune system for combatting COVID-19 [86].

Given the important relationship between probiotics, microbiota, and COVID-19, several researchers directly focused on probiotics that may have a high antiviral effect on COVID-19. Seven clinical trials studying the effects of probiotics on COVID-19 have been reported, with six completed (Table 1). A clinical study of 123 SARS-CoV-2 infected patients with severe symptoms, treated with a mixture of probiotics such as *Lactobacillus acidophilus* and *Bifidobacterium infantis*, showed clinical evidence that probiotics could moderate the immune function and reduce secondary infection [87]. In another study, a team conducted a randomized, placebo-controlled clinical trial to evaluate the efficacy and safety of a novel probiotic formulation in COVID-19 outpatients [88]. Patients aged from 18 to 60 years were treated with a probiotic formulation of three strains of *Lactiplantibacillus plantarum* (KABP022, KABP023, and KAPB033), together with one strain of *Pediococcus acidilactici* KABP021 or were given a placebo, which they took orally once daily for 30 consecutive days. In this study, remission, defined as a negative RT-qPCR and symptom clearance, was assessed. The remission proportion for the probiotic-treated group was at ~53.1%, significantly higher than that of the placebo group (~28.1%). Separately, another study showed that the high consumed quantity of fermented vegetables or cabbage might be associated with a low COVID-19 death rate in some countries in Eastern Asia and Central Europe [89].

## 4. Computational Insight: The Future of Probiotics

Probiotics play an important role in human microbiota eubiosis [90]. The recognition of the human microbiome as a biological system has opened new avenues with the potential to influence health and disease significantly [91]. This includes the paradigm-shift opportunity to better understand the relationship between the host and microbiome environment, facilitated by advances in genomics and bioinformatics, which provide efficient tools to acquire and mine biological and clinical data, such as diet, microbiome (particularly of the gut), and health profile [92]. Therefore, amid the COVID-19 pandemic, applying technology and computational methods as an integral part of probiotics-related research is a high potential strategy against this lethal virus. In this context, we review the available data sources and the currently used computational approach, including the microbiome-driven approach and ensemble-driven docking approach, which could be two potential ways for long-term fighting with SARS-CoV-2. We also provide a case study showing the antiviral activity of *Lactobacillus plantarum* metabolite against SARS-CoV-2 non-structural protein 13 (nsp13) using a molecular docking approach.

### 4.1. Data

Data play an essential role in analysis and evaluation, thereby making appropriate conclusions and offering optimal solutions. Given the accelerated pace in the development of computational tools, the surge in the size of data—probiotics, gut microbiota, human health profile, and diet—presents various opportunities in translating the data into knowledge, delving deeply into the studies in microbiota-related human health [92]. It is evident that gut microbiota plays a crucial role in modulating human health [93], the gut microbiota can be influenced by diet [94]. Paving the possible way for data integration, a list of publicly available data sources is provided in Table 2.

Different approaches have been conducted to collect and create a comprehensive, high-quality data. Probiotics and gut microbiota data with functional characteristics have been identified using metaproteomics and metametabolomics [95,96]. Additionally, gut microbiota metagenomics and metatranscriptomics can also be quantified using the sequencing approach [97,98]. Separately, the human health profile data such as gender, age, height, weight, blood pressure, and disease status can be attained from a questionnaire, interviews, or clinical records [92]. Similarly, the diet database is often built using a questionnaire approach, either in subjective reports or through objective observation. One of the dietary assessment methods for self-reporting is using food frequency questionnaires (FFQ), which has been widely utilized since the 1990s [99]. The intake amount of metabolites and nutrients such as protein, fat, vitamin, and minerals are calculated by checking the consumed food and food composition database. Notably, it is also feasible to characterize the microbiota of intake food using metagenomic sequencing [92].

**Table 2:** Available data of human gut microbiota, health profile, and diet (all mentioned URLs were accessed on 28 November 2021).

Category	Database or Project Name	URL	Reference
Probiotics	Probiotic Strain Database	https://microbiomepost.com/probiotic-strain-database	-
	Probiotics Database	https://www.optibacprobiotics.com/professionals/probiotics-database	-
	PBDB	http://gsbios.com/index/experimental/pbdben?id=7	-
Gut microbiota	NIH Human Microbiome Project	https://hmpdacc.org/hmp/	[100]
	gutMDisorder	http://bio-annotation.cn/gutMDisorder/	[101]
	Amadis	http://gift2disease.net/GIFTED/	[102]
	HumGut	http://arken.nmbu.no/~larssn/humgut/	[103]
	MANTA	https://mizuguchilab.org/manta/	[104]
	GutFeeling KnowledgeBase	https://hive.biochemistry.gwu.edu/gfkb	[105]
Human health profile	CDC WONDER	https://wonder.cdc.gov/	[106]
	1000IBD Project	https://1000ibd.org/	[107]
	HealthMap	https://www.healthmap.org/en/	[108]
Diet	Global Dietary Database	https://www.globaldietarydatabase.org/	-
	FAOSTAT	http://www.fao.org/faostat/en/	-
	Diet Compositions	https://ourworldindata.org/diet-compositions	-
	CABI—Nutrition and Food Sciences	https://www.cabi.org/publishing-products/nutrition-and-food-sciences-database/	-

### 4.2. Technology: Microbiome-Driven Approach

To date, probiotics and their application have been tremendously reported for their use to prevent or treat various intestinal diseases such as antibiotic-associated diarrhea, inflammatory bowel disease, and Crohn’s disease [109]. Moreover, probiotics could modulate and restore the gut microbiome composition [90]. Hence, using probiotics for COVID-19 patients, research how gut microbiome affecting the SARS-CoV-2 could be a potential pathway in this area. The gut microbiome comprises of trillions of bacteria and other microbes [110]. The gut microbiome plays a vital role in health maintenance, disease pathogenesis, immune homeostasis, and host energy metabolism [111]. Due to the gut-lung axis, the gut microbiome may have an impact on respiratory tract infections, and may be involved in the magnitude of SARS-CoV-2 severity [68]; hence, with the ongoing COVID-19 pandemic, the SARS-CoV-2 infected patient’s gut microbiome merits attention and research. Notably, the field of microbiome-driven approach associated with computational methods could be utilized to facilitate such research of the gut microbiome. Various bioinformatics tools and pipelines are available for the study of the gut microbiome research in the context of metagenomics and metatranscriptomics (Table 3), which could provide insights into the interaction between gut microbiota and viral infections. Tools for whole metagenomic sequencing are MetaPhlAn2 [112], MG-RAST [113], MEGAHIT [114], HUMAnN2 [115], QIIME [116], mothur [117] and SPAdes [118]; while for metatranscriptomics, tools such as SOAPdenovo [119], SAMSA2 [120] and mOTUs2 [121] are used.

It has been reported that the severity of COVID-19 symptoms might be associated with the differences in the gut microbiota of each person. Metagenomics sequencing carried fecal DNA samples obtained from COVID-19 patients, resulting in a significant difference between gut microbiome of healthy controls and patients, particularly for some gut commensals such as *Faecalibacterium prausnitzii* and *bifidobacteria*, which were underrepresented in COVID-19 patients [68]. This reduction of microbiota remained for around 30 days after viral clearance. In a pilot study of 15 COVID-19 patients in Hong Kong, shotgun metagenomic sequencing was conducted to analyze fecal samples from them [71]. The microbiome data were compared with six community-acquired pneumonia (CAP) patients and 15 healthy controls. The profiling of microbiota was implemented using MetaPhlAn2 (V2.9). From the obtained results, the researchers concluded that there were alterations in the fecal microbiome during the hospitalization time in COVID-19 patients, compared with healthy individuals. Furthermore, they proposed that if there are effective strategies to improve the intestinal microbiota, it may decrease SARS-CoV-2 severity [71]. As for the metatranscriptomics study, the analysis was done on 62 COVID-19 patients and 125 non-COVID-19 pneumonia patients [122]. Three transcriptional aspects, including the microbiome, pathogens, and host responses, were assessed. Results showed that COVID-19 patients had a more disrupted airway microbiome compared with non-COVID-19 patients. Metatranscriptome sequencing was also implemented for the bronchoalveolar lavage fluid in the eight COVID-19 patients, 25 patients with CAP, and 20 healthy individuals [123]. The results indicated that the lung microbiota in COVID-19 patients was similar to those in CAP patients. Despite not directly involving the gut microbiome, this study showed the potential of the metatranscriptomics method, primarily when used to study the gut microbiota of COVID-19 patients.

### 4.3. Technology: Ensemble-Driven Docking Approach

A traditional drug discovery process is time-consuming, taking up to around 10–15 years for a drug to be approved and available in the market [124]. Applying computer-aided drug discovery (CADD) could improve the effectiveness and efficiency of drug research and bring a high benefit in saving both cost and time [125]. To date, this field has been developing and becoming an integral part of drug discovery projects, along with the development of computational algorithms, bioinformatics tools, and the availability of a wide range of protein structures and pharmaceutical compounds [126]. Different studies have highlighted the role, importance, advanced applications, and the challenges of computational approaches [127]. Significantly, the successful applications of CADD in antiviral drug design have been reported, in which viral protein targets are focused [128,129,130]. The characteristics of viral targets could be elucidated by the modeling, simulation, and predicting conformational, physicochemical properties. Moreover, the interaction and binding affinity between viral proteins and pharmaceutical compounds or inhibitors could be identified by using computational techniques. Additionally, the virtual screening and structure-activity relationship between viral targets and ligands have also been implemented [128]. Regarding the application of CADD in antiviral-related researches, bioinformatics has been widely utilized to identify consistent drugs for different infectious diseases such as dengue, influenza, and Zika [131,132,133]. To date, bioinformatics has been marked at different milestones, including analyzing virus origin and its evolution, genome sequencing, modeling structural biological entities, researching host genetic susceptibility [134].

Using computational approaches can speed up discovering the therapeutic strategy against COVID-19. In fact, with this exact purpose, COVID-19-related data has been shared worldwide with unprecedented speed. The bioinformatics approaches in the process of drug discovery, such as modeling, molecular docking, molecular dynamics simulation, and in silico ADMET study, have been researched and applied to screen the potential compounds (both antiviral agents and probiotics) for combatting COVID-19 from numerous databases (Table 4). Furthermore, analyzing SARS-CoV-2 data by bioinformatics could also lead to valuable information in both fundamental and applied science. Studying the effect of probiotics on SARS-CoV-2 by using computational techniques could be potential and paving the way for further antiviral probiotics research.

In the context of a low quantity of known compounds against SARS-CoV-2 activity, the drug development strategy based upon molecular structure has been focused on. Especially, molecular docking is one of the main techniques that contribute effective ways to explore antiviral agents for SARS-CoV-2. Molecular docking is a method for predicting the binding orientation interaction between a particular ligand and an active site of the receptor [135]. The more negative binding energy, the better the docking score. Moreover, some types of interactions such as hydrogen, ionic, and Van der Waals should also be considered. Based on results of molecular docking, it is possible to choose hit compounds for targeted viral proteins. Several databases of virtual chemical compound libraries are publicly available in the process of exploring hit compounds, such as PubChem [136] and ZINC [137], among others. Some popular molecular docking programs are AutoDock [138], AutoDock Vina [139], FlexX [140], and GLIDE [141].

**Figure 3:**
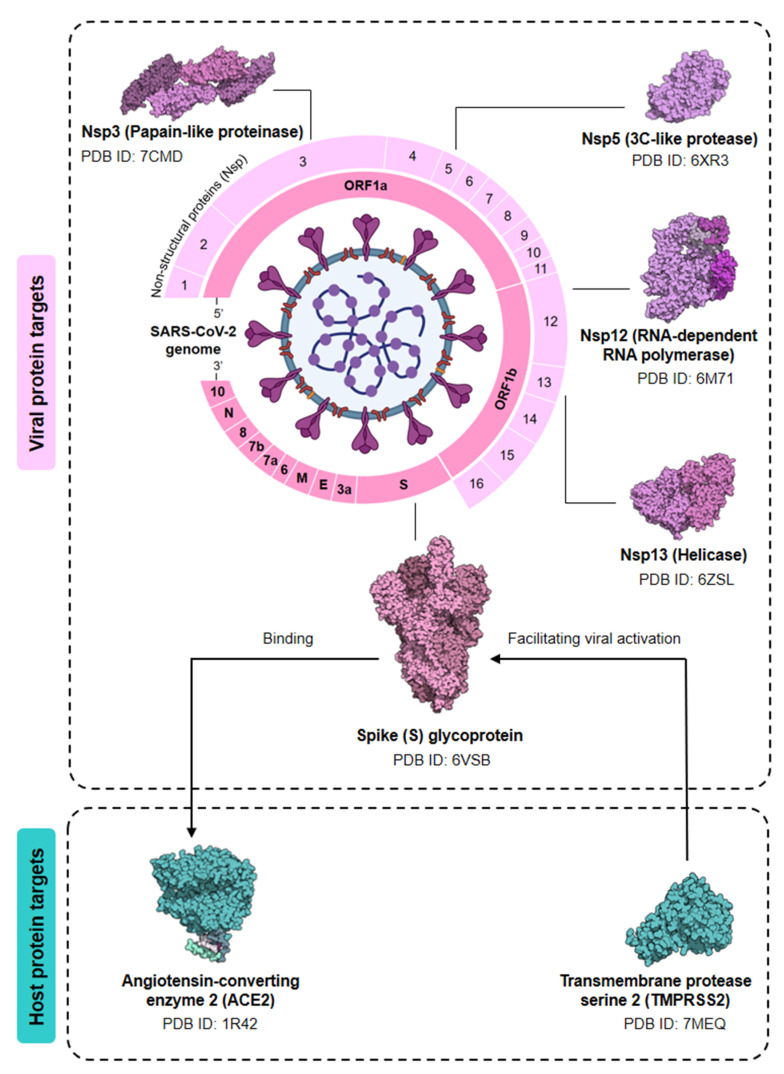
The structures of possible viral and host protein targets could be inhibited by probiotic metabolites to prevent SARS-CoV-2. Angiotensin-converting enzyme 2 (ACE2), which locates on host cells, is the primary cell entry receptor for SARS-CoV-2 [174]; transmembrane protease serine 2 (TMPRSS2), which facilitate viral activation, is a cell surface protein expressed in the respiratory and GI tract [175]. SARS-CoV-2 requires both ACE2 and TMPRSS2 for entry into cells [176]. Spike (S) protein involves mainly in the receptor recognition and viral entry of SARS-CoV-2 [177]; Papain-like proteinase (PL^pro^) has an essential role in viral polyprotein cleavage and maturation [178]; 3C-like main protease (3CL^pro^) plays a key role in control viral replication [179]; RNA-dependent RNA polymerase (RdRp), a viral enzyme, involves in viral RNA replication in host cells [180]; Nsp13 is a helicase requiring adenosine triphosphate (ATP) to translocate and unwind SARS-CoV-2 RNA [181].

Some possible SARS-CoV-2 protein targets have been reported (Figure 3). A plethora of studies applied molecular docking to study the inhibitory effects of compounds on SARS-CoV-2. One of the studies showed various potential hits for five different protein targets of COVID-19, including the coronavirus 3C-like protease (3CL^pro^), RNA dependent RNA polymerase (RdRp), angiotensin-converting enzyme 2 (ACE2), papain-like protease (PL^pro^), and spike glycoprotein-receptor binding domain (SGp-RBD), based on the results of docking scores, in silico ADMET properties, drug-likeness properties, and protein–ligand interaction study [142]. As many as seven potential phytochemicals, including obacunone, corosolic acid, 7-deacetyl-7-benzoylgedunin, limonin, ursolic acid, glycyrrhizic acid, and maslinic acid, were considered to be sufficiently used to formulate a further optimization in drug design to fight against COVID-19. Another molecular docking study depicted the effect of tea polyphenols, including epigallocatechin (EGCG) from green tea and theaflavin digallate (TF3) from black tea [135]. The docking results showed that these two tea polyphenols have potential activity in treating SARS-CoV-2. Additionally, by applying molecular docking, punicalagin, and punicalin, the constituents of pomegranate peel extract, were shown to have potential effects for the interaction with protein targets of SARS-CoV-2 ACE2, spike glycoprotein, furin, and transmembrane serine protease [143]. Moreover, the binding of luteolin, ribavirin, chloroquine, and remdesivir with 3CL^pro^, PL^pro^, RdRp, and spike proteins of SARS-CoV-2 were conducted by computational approaches [144]. It was shown that luteolin, the main flavonoid of honeysuckle, could bind with high affinity to the active sites of the main protease of the virus, implying a potential antiviral activity.

Although several clinical trials have been conducted to assess symptoms and patients’ health conditions, the molecular mechanism of these effects has remained elusive. Until now, there have been a few studies involved in the impact of *Lactobacillus* metabolites on inhibiting SARS-CoV-2. In a study, Anwar and colleagues studied the effect of different probiotics including plantaricin W, plantaricin BN, plantaricin JLA-9, plantaricin D from *Lactobacillus* on blocking a residual binding protein on SARS-CoV-2 spike protein and the interaction of spike protein with human ACE2 receptor by using docking and molecular dynamics simulation method [145]. The results have shown that plantaricin molecules may have the blocking effect for SARS-CoV-2 RdRp enzyme and spike protein. A similar approach was reported by Balmeh and colleagues where sequence manipulation of metabolite, namely glycocin F from *Lactobacillus* were used to investigate the potential binding towards SARS-CoV-2 drug targets [146]. The homology modeling and molecular docking methods were also used to assess the effects of plantaricin E and plantaricin F from *Lactobacillus plantarum* Probio-88 on SARS-CoV-2 helicase [147]. The formation of hydrogen bonding and high binding affinity indicated that the association of both plantaricin on viral helicase might serve as a blocker by preventing the binding of ssRNA on helicase, paving the way for deeper research in probiotic metabolites against SARS-CoV-2.

**Table 4:** Relevant computational methods and bioinformatics tools to research antiviral agents and probiotics against SARS-CoV-2.

No.	Aim of Research	Computational Methods	Tools [Reference of the Tools]	Reference
1	Investigate and identify potential hits that could inhibit SARS-CoV-2 by carrying out virtual screening, which included molecular docking, in silico ADMET, and simulation	Screened phytochemicals against five protein targets of COVID-19 (3CL^pro^, RdRp, ACE2, PL^pro^, SGp-RBD) Predicted best-docked score phytochemicals in terms of: in silico ADMET prediction Drug-likeness prediction	- AutoDock Vina [139] - pkCSM [148] - Molinspiration [149]	[142]
2	Research the role of tea polyphenols on SARS-CoV-2 inhibition	- Ligand preparation - Binding site prediction - Molecular docking - Mutagenesis analysis - Evaluate the stability of mutant protein structure - Molecular dynamics simulation - Generate ligand topology files - Molecular visualizatio	- AutoGridFR [150] - AutoDock Vina [139] - Mutagenesis wizard [151] - DynaMut web server [152] - CHARMM-GUI web server [153] - Visual Molecular Dynamics (VMD) [154]	[135]
3	Study the interaction of luteolin, ribavirin, chloroquine, and remdesivir with the main protease of COVID-19	- Molecular docking	- AutoDock Vina [139]	[144]
4	Investigate the effects of pomegranate peel extract on SARS-CoV-2 spike glycoproteins, furin, ACE2, and transmembrane serine protease 2	- Protein active site prediction - Molecular docking - Analyze the best binding affinity docking positions with a visualization tool	- DoGSiteScorer [155] - AutoDock Vina [139] - Discovery Studio [156]	[143]
5	Investigate the effect of remdesivir, sofosbuvir, ribavirin, galidesivir and tenofovir on RdRp	- Homology model for RdRp - Examining the model - Checking the validity of the model - Optimizing the model - Molecular docking - Examining the structure after docking	- SWISS-MODEL server [157] - MolProbity web server [158] - PROCHECK [159]; Verify 3D [160]; ERRAT [161] - AutoDock Vina [139] - Protein–Ligand Interaction Profiler (PLIP) webserver [162]	[163]
6	Test several anti-polymerase drugs against SARS-CoV-2 RdRp by using computational approaches	- Homology modeling - Evaluating chemical properties, bonds, and angles of RdRp - Molecular docking - Toxicity validation and AdmetSAR profiling	- MODELLER [164] - Molecular Operating Environment (MOE) software [133] - AdmetSAR online tool [165]	[166]
7	Investigate the effect of grazoprevir (antiviral drug against HCV) on SARS-CoV-2 by using in silico methods	- Protein selection and prediction - Ligand selection and preparation - Molecular docking - Image generation and protein–ligand analysis - Molecular dynamics simulation	- UCSF Chimera [167] - SWISS-MODEL server [157] - AutoDock 4.2 [168] - Lig-Plot + [169] - GROMACS [170], GROMOS 54A7 force field [171]	[172]
8	Investigate the effect of probiotics (Plantaricin JLA-9, Plantaricin W, Plataricin D) on spike protein and the interaction of spike protein with human ACE2 receptor	- Protein modeling - Generating model quality parameters - Ligand preparation - Ligand protein interaction and generation of images - Molecular dynamics simulations; visualizing the graphs of Root Mean Square Deviation (RMSD)	- SWISS-MODEL server [157] - Molecular Operating Environment (MOE) software [133] - Discovery studio, UCSF Chimera package [167], and PLIP web server [162] - GROMACS [170]	[145]
9	Investigate the action of probiotic *Lactobacillus plantarum* Probio-88 against SARS-CoV-2 replication and immune regulation, with in silico study of metabolite Plantaricin E (PlnE) and Plantaricin F (PlnF) from *Lactobacillus plantarum* Probio-88	- Molecular docking	- SWISS-MODEL server [157] - HADDOCK 2.4 [173] - Visual Molecular Dynamics (VMD) [154]	[147]

### 4.4. Case Study: Metabolites of Lactobacillus Plantarum as a Promise to COVID-19

*Lactobacillus plantarum* metabolites have been proved to be potential against SARS-CoV-2 [145,147]. Among the viral protein targets, helicase nsp13, a non-structural protein highly conserved among coronavirus species, plays a crucial role in SARS-CoV-2 replication and is considered an excellent antiviral target [182,183]. Notably, plantaricin (PlnE and PlnF) from *Lactobacillus plantarum* Probio-88 showed the potential of antiviral activity in SARS-CoV-2 by binding at either ssRNA or ATP binding sites of helicase [147]. The binding of PlnE and PlnF were interacted and analyzed individually with helicase. However, Kristiansen and co-workers had shown that the PlnE and PlnF might interact in an antiparallel manner, and their functions depend on complementary activity [184,185]. Similarly, it was also reported that PlnE and PlnF form a helix bundle and lie in a staggered fashion [186]. Apart from the structural concern, PlnEF was suggested to be present in pairs in order to synergy with antibiotics against bacteria [187]. These findings were refuted with the experimental design in the previous study. Therefore, the study is extended further where the PlnE and PlnF sequences were used to reconstruct the 3D model as a single structure.

**Figure 4:**
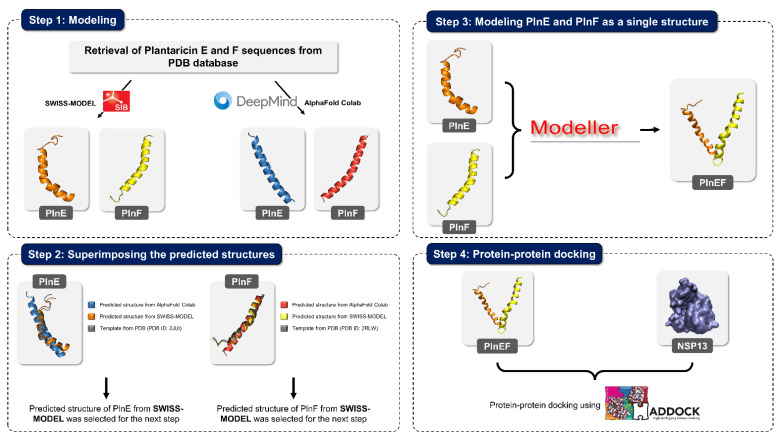
Schematic representation for the reconstruction of PlnE and PlnF. The sequence of PlnE and PlnF were separately modeled using SWISS-MODEL server and AlphaFold Colab, followed by superimposing predicted structures with PlnE template (PDB ID: 2JUI) and PlnF template (PDB ID: 2RLW). The SWISS-MODEL predicted structures were chosen for further modeling due to the lower RMSD value with the templates compared to AlphaFold Colab predicted structures. A homology modeling approach was used to rebuild PlnE and PlnF as a single structure using MODELLER v10.1. The best structure was used to dock against SARS-CoV-2 helicase nsp13 using the protein-protein docking approach.

In this case study, prior to modeling PlnEF as a whole structure, AlphaFold Colab [188] was utilized to generate the predicted structures of PlnE and PlnF separately (Figure 4). AlphaFold Colab, a Google Colab notebook allowing users to predict protein structure, is a simplified version of AlphaFold v2.1.0, which has been developed by DeepMind [188,189]. The predicted structures of PlnE and PlnF derived from AlphaFold Colab, and SWISS-MODEL were superimposed with the template using PyMOL [190]. The RMSD value between AlphaFold Colab structure and PlnE template (PDB ID: 2JUI) was 2.91 Å, while SWISS-MODEL was 0.62 Å (Table 5). Similarly, the RMSD value for AlphaFold Colab and PlnF template (PDB ID: 2RLW) was 1.75 Å; for SWISS-MODEL and template, this value reached 0.33 Å. Due to the smaller RMSD value, the predicted structures of PlnE and PlnF from SWISS-MODEL were selected for further modeling as a single structure (Figure 4). The higher RMSD between predicted structures from AlphaFold Colab and templates could be elucidated. The Colab notebook uses no templates or homologous structures, leading to the higher discrepancy between obtained results and templates [188].

**Figure 5:**
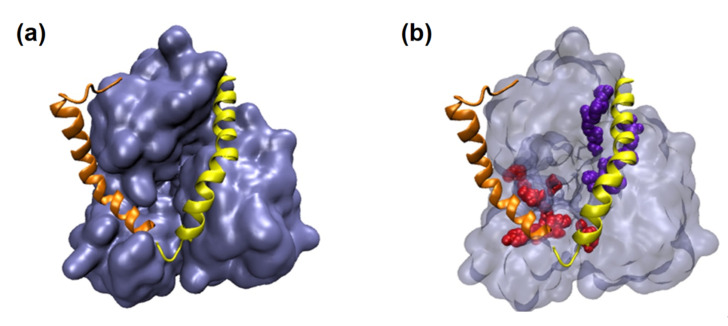
Molecular docking of PlnEF towards SARS-CoV-2 helicase nsp13. PlnE (orange) and PlnF (yellow) were modeled as a single structure using MODELLER v10.1. (**a**) PlnEF was potentially bound toward the incision of the ssRNA and ATP binding site. (**b**) ssRNA and ATP binding are red (Ser485, Lys146, Lys139, Tyr180, His230, Tyr198, Arg212, Pro335, Arg339, Asn516) and violet (Glu537, Arg567, Arg443, His290, Arg442, Asn265, Gly439, Lys288), respectively.

PlnE and PlnF’s predicted structure was modeled as a single structure using MODELLER v10.1 [191]. The built model of PlnEF consists of a helix bundle in an antiparallel manner (Figure 4). A total of 30 structures were made, and the best structure was determined based on the shortest distance between the C and N-terminal of PlnE and PlnF. Subsequently, both built models were docked towards SARS-CoV-2 helicase nsp13 (PDB ID: 6ZSL) using HADDOCK 2.4 [173]. Despite the HADDOCK scoring, binding conformation was used to determine the binding potential for PlnE and PlnF towards SARS-CoV-2 helicase. Among the three top clusters from HADDOCK clustering results, one of the docked results signified the critical possibility of PlnE and PlnF toward both ATP and ssRNA binding site of SARS-CoV-2 helicase nsp13 (Figure 5a) with the binding affinity of −19.3 kcal/mol. The binding strength for PlnEF toward SARS-CoV-2 helicase nsp13 is −1.9 kcal/mol and −3.4 kcal/mol stronger than PlnE and PlnF, which docked separately [147]. PlnE and PlnF are energetically favorable toward opening the cavity at both ATP and ssRNA binding sites (Figure 5b). Besides complementing each other structurally, they might produce a synergistic effect which might strengthen the binding affinity. This further supports our postulation earlier [147], in which PlnE and PlnF may potentially serve as the blocker to prevent the binding of the ATP and ssRNA.

## 5. Conclusions

Battling with COVID-19 is still a long way to go. The number of COVID-19 cases increases daily, despite available vaccines and many campaigns to immunize against SARS-CoV-2. This is placing heavy burdens on both human health and the economy. In this circumstance, utilizing probiotics as a complementary strategy besides vaccines to inhibit COVID-19 should be considered due to the postulated antiviral effects of probiotics and their metabolites. Additionally, the molecular mechanisms of probiotics can provide new insights into how probiotics combat SARS-CoV-2 infection. By exploiting significant advances in bioinformatics and computational studies, unraveling the molecular actions of probiotics on SARS-CoV-2 is feasible. Currently available data on probiotics, human microbiota, health profile, and diet can be used as valuable sources in part of probiotics-related research against viruses, including SARS-CoV-2, along with two well-known computational approaches, microbiome-driven approach and ensemble-driven docking approach. The case study that we researched and provided discloses the antiviral potential of *Lactobacillus plantarum* metabolite PlnE and PlnF against SARS-CoV-2 nsp13 using molecular docking method is an example proving the possibility of studying molecular insight of probiotics against COVID-19. Therefore, integrating probiotic data with existing computational tools will significantly benefit COVID-19 research.

## Figures and Tables

**Table 1:** Clinical trials on the effect of consuming probiotics against COVID-19. The data is up-to-date as of 28 November 2021, retrieved from https://clinicaltrials.gov/. The search query was “condition or disease” = “covid19”, “other terms” = “probiotics.”.

No.	Identifier	Title	Treatment	Probiotic Strain	Number Enrolled	Status
1	NCT04517422	Efficacy and safety of *Lactobacillus plantarum* and *Pediococcus acidilactici* as co-adjuvant therapy for reducing the risk of severe disease in adults with SARS-CoV-2 and its modulation of the fecal microbiota: A randomized clinical trial	Once per day, administered for 30 days	Combination of 4 probiotic strains, including 3 *Lactobacillus plantarum* strains CECT30292, CECT7484, CECT7485, and *Pediococcus acidilactici* strain CECT7483	300	Completed
2	NCT04458519	Clinical study of efficacy of intranasal probiotic treatment to reduce severity of symptoms in COVID-19 infection	Twice per day, administered for 14 days	*Lactococcus lactis* W136	23	Completed
3	NCT04854941	Efficacy of probiotics (*Lactobacillus rhamnosus*, *Bifidobacterium bifidum*, *Bifidobacterium longum* subsp. *infantis* and *Bifidobacterium longum*) in the treatment of hospitalized patients with novel coronavirus infection	3 times per day, administered for 14 days	Combination of 4 probiotic strains, including *Lactobacillus rhamnosus* PDV 1705, *Bifidobacterium bifidum* PDV 0903, *Bifidobacterium longum* subsp. *infantis* PDV 1911 and *Bifidobacterium longum* PDV 2301	200	Completed
4	NCT04399252	A randomized trial of the effect of *Lactobacillus* on the microbiome of household contacts exposed to COVID-19	2 capsules per day, administered for 28 days	*Lactobaciltus rhamnosus* GG	182	Completed
5	NCT04734886	Exploratory study for the probiotic supplementation effects on SARS-CoV-2 antibody response in healthy adults	2 capsules per day, administered for 6 months	*Lactobacillus reuteri* DSM 17938	161	Completed
6	NCT05043376	A randomized, open-label, and controlled clinical trial to study the adjuvant treatment benefits of probiotic *Streptococcus salivarius* K12 to prevent/reduce lung inflammation in mild-to-moderate hospitalized patients with COVID-19	2 tablets per day, administered for up to 14 days	*Streptococcus salivarius* K12	50	Completed
7	NCT04366180	Multicentric study to assess the effect of consumption of *Lactobacillus coryniformis* K8 on healthcare personnel exposed to COVID-19	Once per day, administered for 8 weeks	*Lactobacillus coryniformis* K8	314	Ongoing

**Table 3:** List of bioinformatics tools for studying the gut microbiome via methods including metagenomics and metatranscriptomics (all mentioned URLs were accessed on 28 November 2021).

Method	Description	Tools	URL	Reference
Metagenomics	Metagenomics is the culture-independent analysis of a collection of genomes from microbial communities contained in natural living environments.	MetaPhlAn2	https://huttenhower.sph.harvard.edu/metaphlan2/	[110]
		MG-RAST	https://www.mg-rast.org/	[113]
		MEGAHIT	https://github.com/voutcn/megahit	[114]
		HUMAnN2	https://huttenhower.sph.harvard.edu/humann2/	[115]
		QIIME	http://qiime.org/	[116]
		mothur	https://mothur.org/	[117]
		SPades	https://cab.spbu.ru/software/spades/	[118]
Metatranscriptomics	Metatranscriptomics, also a culture-independent method, allows studying of the expressed RNA transcripts in the microbiome.	SOAPdenovo	http://soapdenovo2.sourceforge.net/	[119]
		SAMSA2	https://github.com/transcript/samsa2	[120]
		mOTUs2	https://motu-tool.org/ https://github.com/motu-tool/mOTUs	[121]

**Table 5:** RMSD values of predicted structures of PlnE and PlnF modeled by AlphaFold Colab and SWISS-MODEL, in comparing with each other or with templates.

	Superimpose	RMSD (Å)
PlnE	AlphaFold Colab/SWISS-MODEL	3.07
	AlphaFold Colab/Template	2.91
	SWISS-MODEL/Template	0.62
PlnF	AlphaFold Colab/SWISS-MODEL	1.48
	AlphaFold Colab/Template	1.75
	SWISS-MODEL/Template	0.33

## Data Availability

Public data was used. Information related to the data is provided in the main text.

## References

[1] Gupta S.D. (2020). Coronavirus Pandemic: A Serious Threat to Humanity. J. Health Manag..

[2] Anderson R.M., Vegvari C., Hollingsworth T.D., Pi L., Maddren R., Ng C.W., Baggaley R.F. (2021). The SARS-CoV-2 pandemic: Remaining uncertainties in our understanding of the epidemiology and transmission dynamics of the virus, and challenges to be overcome. Interface Focus.

[3] Meredith S., Towey R. (2021). WHO Labels New COVID Strain, Named Omicron, a ‘Variant of Concern,’ Citing Possible Increased Reinfection Risk.

[4] Cascella M., Rajnik M., Aleem A., Dulebohn S.C., Di Napoli R. (2021). Features, Evaluation, and Treatment of Coronavirus (COVID-19). StatPearls.

[5] Gao Z., Xu Y., Sun C., Wang X., Guo Y., Qiu S., Ma K. (2021). A systematic review of asymptomatic infections with COVID-19. J. Microbiol. Immunol. Infect..

[6] Jiang F., Deng L., Zhang L., Cai Y., Cheung C.W., Xia Z. (2020). Review of the Clinical Characteristics of Coronavirus Disease 2019 (COVID-19). J. Gen. Intern. Med..

[7] Shivakumar S., Smibert O.C., Trubiano J.A., Frauman A.G., Liew D.F. (2020). Immunosuppression for COVID-19: Repurposing medicines in a pandemic. Aust. Prescr..

[8] Chen Y., Gu S., Chen Y., Lu H., Shi D., Guo J., Wu W.R., Yang Y., Li Y., Xu K.J. (2021). Six-month follow-up of gut microbiota richness in patients with COVID-19. Gut.

[9] Xu K., Cai H., Shen Y., Ni Q., Chen Y., Hu S., Li J., Wang H., Yu L., Huang H. (2020). Management of corona virus disease-19 (COVID-19): The Zhejiang experience. J. Zhejiang Univ. Med. Sci..

[10] Yahfoufi N., Mallet J.F., Graham E., Matar C. (2018). Role of probiotics and prebiotics in immunomodulation. Curr. Opin. Food Sci..

[11] Plaza-Díaz J., Ruiz-Ojeda F.J., Vilchez-Padial L.M., Gil A. (2017). Evidence of the Anti-Inflammatory Effects of Probiotics and Synbiotics in Intestinal Chronic Diseases. Nutrients.

[12] Wang Y., Wu Y., Wang Y., Xu H., Mei X., Yu D., Wang Y., Li W. (2017). Antioxidant Properties of Probiotic Bacteria. Nutrients.

[13] Al Kassaa I. (2017). Antiviral Probiotics: A New Concept in Medical Sciences. New Insights on Antiviral Probiotics: From Research to Applications.

[14] Mack D. (2005). Probiotics-mixed messages. Can. Fam. Physician Med. Fam. Can..

[15] Bezirtzoglou E., Stavropoulou E. (2011). Immunology and probiotic impact of the newborn and young children intestinal microflora. Anaerobe.

[16] Marinova V.Y., Rasheva I.K., Kizheva Y.K., Dermenzhieva Y.D., Hristova P.K. (2019). Microbiological quality of probiotic dietary supplements. Biotechnol. Biotechnol. Equip..

[17] Salminen S.J., Gueimonde M., Isolauri E. (2005). Probiotics that modify disease risk. J. Nutr..

[18] Mousavi Khaneghah A., Abhari K., Eş I., Soares M.B., Oliveira R.B.A., Hosseini H., Rezaei M., Balthazar C.F., Silva R., Cruz A.G. (2020). Interactions between probiotics and pathogenic microorganisms in hosts and foods: A review. Trends Food Sci. Technol..

[19] Wan L.Y., Chen Z.J., Shah N.P., El-Nezami H. (2016). Modulation of Intestinal Epithelial Defense Responses by Probiotic Bacteria. Crit. Rev. Food Sci. Nutr..

[20] Villena J., Shimosato T., Vizoso-Pinto M.G., Kitazawa H. (2020). Editorial: Nutrition, Immunity and Viral Infections. Front. Nutr..

[21] Boutayeb A. (2010). The Burden of Communicable and Non-Communicable Diseases in Developing Countries. Handbook of Disease Burdens and Quality of Life Measures.

[22] Rubin R. (2021). COVID-19 Vaccines vs Variants—Determining How Much Immunity Is Enough. JAMA.

[23] Lopez Bernal J., Andrews N., Gower C., Gallagher E., Simmons R., Thelwall S., Stowe J., Tessier E., Groves N., Dabrera G. (2021). Effectiveness of COVID-19 Vaccines against the B.1.617.2 (Delta) Variant. N. Engl. J. Med..

[24] Ogunrinola G.A., Oyewale J.O., Oshamika O.O., Olasehinde G.I. (2020). The Human Microbiome and Its Impacts on Health. Int. J. Microbiol..

[25] Wang B., Yao M., Lv L., Ling Z., Li L. (2017). The Human Microbiota in Health and Disease. Engineering.

[26] Rajput S., Paliwal D., Naithani M., Kothari A., Meena K., Rana S. (2021). COVID-19 and Gut Microbiota: A Potential Connection. Indian J. Clin. Biochem. IJCB.

[27] Rodriguez W.J., Kim H.W., Brandt C.D., Bise B., Kapikian A.Z., Chanock R.M., Curlin G., Parrott R.H. (1980). Rotavirus gastroenteritis in the Washington, DC, area: Incidence of cases resulting in admission to the hospital. Am. J. Dis. Child..

[28] Tate J.E., Burton A.H., Boschi-Pinto C., Parashar U.D. (2016). Global, Regional, and National Estimates of Rotavirus Mortality in Children <5 Years of Age, 2000–2013. Clin. Infect. Dis. Off. Publ. Infect. Dis. Soc. Am..

[29] Ma C., Wu X., Nawaz M., Li J., Yu P., Moore J.E., Xu J. (2011). Molecular characterization of fecal microbiota in patients with viral diarrhea. Curr. Microbiol..

[30] Preidis G.A., Saulnier D.M., Blutt S.E., Mistretta T.A., Riehle K.P., Major A.M., Venable S.F., Barrish J.P., Finegold M.J., Petrosino J.F. (2012). Host response to probiotics determined by nutritional status of rotavirus-infected neonatal mice. J. Pediatr. Gastroenterol. Nutr..

[31] Pant N., Marcotte H., Brüssow H., Svensson L., Hammarström L. (2007). Effective prophylaxis against rotavirus diarrhea using a combination of Lactobacillus rhamnosus GG and antibodies. BMC Microbiol..

[32] Qiao H., Duffy L.C., Griffiths E., Dryja D., Leavens A., Rossman J., Rich G., Riepenhoff-Talty M., Locniskar M. (2002). Immune responses in rhesus rotavirus-challenged BALB/c mice treated with bifidobacteria and prebiotic supplements. Pediatric Res..

[33] Muñoz J.A., Chenoll E., Casinos B., Bataller E., Ramón D., Genovés S., Montava R., Ribes J.M., Buesa J., Fàbrega J. (2011). Novel probiotic Bifidobacterium longum subsp. infantis CECT 7210 strain active against rotavirus infections. Appl. Environ. Microbiol..

[34] Shu Q., Qu F., Gill H.S. (2001). Probiotic Treatment Using Bifidobacterium lactis HN019 Reduces Weanling Diarrhea Associated with Rotavirus and Escherichia coli Infection in a Piglet Model. J. Pediatric Gastroenterol. Nutr..

[35] Liu F., Li G., Wen K., Wu S., Zhang Y., Bui T., Yang X., Kocher J., Sun J., Jortner B. (2013). Lactobacillus rhamnosus GG on rotavirus-induced injury of ileal epithelium in gnotobiotic pigs. J. Pediatr. Gastroenterol. Nutr..

[36] Ang L., Arboleya S., Lihua G., Chuihui Y., Nan Q., Suarez M., Solís G., de los Reyes-Gavilán C.G., Gueimonde M. (2014). The establishment of the infant intestinal microbiome is not affected by rotavirus vaccination. Sci. Rep..

[37] García-López R., Pérez-Brocal V., Diez-Domingo J., Moya A. (2012). Gut microbiota in children vaccinated with rotavirus vaccine. Pediatric Infect. Dis. J..

[38] Zhang W., Azevedo M., Wen K., Gonzalez A., Saif L., Li G., Yousef A., Yuan L. (2008). Probiotic Lactobacillus acidophilus enhances the immunogenicity of an oral rotavirus vaccine in gnotobiotic pigs. Vaccine.

[39] Vlasova A.N., Chattha K.S., Kandasamy S., Liu Z., Esseili M., Shao L., Rajashekara G., Saif L.J. (2013). Lactobacilli and bifidobacteria promote immune homeostasis by modulating innate immune responses to human rotavirus in neonatal gnotobiotic pigs. PLoS ONE.

[40] Lehtoranta L., Pitkäranta A., Korpela R. (2014). Probiotics in respiratory virus infections. Eur. J. Clin. Microbiol. Infect. Dis. Off. Publ. Eur. Soc. Clin. Microbiol..

[41] Jung Y.J., Lee Y.T., Ngo V.L., Cho Y.H., Ko E.J., Hong S.M., Kim K.H., Jang J.H., Oh J.S., Park M.K. (2017). Heat-killed Lactobacillus casei confers broad protection against influenza A virus primary infection and develops heterosubtypic immunity against future secondary infection. Sci. Rep..

[42] Smith A.M., Smith A.P. (2016). A Critical, Nonlinear Threshold Dictates Bacterial Invasion and Initial Kinetics During Influenza. Sci. Rep..

[43] Tate M.D., Schilter H.C., Brooks A.G., Reading P.C. (2011). Responses of mouse airway epithelial cells and alveolar macrophages to virulent and avirulent strains of influenza A virus. Viral Immunol..

[44] Harata G., He F., Hiruta N., Kawase M., Kubota A., Hiramatsu M., Yausi H. (2010). Intranasal administration of Lactobacillus rhamnosus GG protects mice from H1N1 influenza virus infection by regulating respiratory immune responses. Lett. Appl. Microbiol..

[45] Izumo T., Maekawa T., Ida M., Noguchi A., Kitagawa Y., Shibata H., Yasui H., Kiso Y. (2010). Effect of intranasal administration of Lactobacillus pentosus S-PT84 on influenza virus infection in mice. Int. Immunopharmacol..

[46] Hori T., Kiyoshima J., Shida K., Yasui H. (2001). Effect of intranasal administration of Lactobacillus casei Shirota on influenza virus infection of upper respiratory tract in mice. Clin. Diagn. Lab. Immunol..

[47] Yeo J.M., Lee H.J., Kim J.W., Lee J.B., Park S.Y., Choi I.S., Song C.S. (2014). Lactobacillus fermentum CJL-112 protects mice against influenza virus infection by activating T-helper 1 and eliciting a protective immune response. Int. Immunopharmacol..

[48] Kikuchi Y., Kunitoh-Asari A., Hayakawa K., Imai S., Kasuya K., Abe K., Adachi Y., Fukudome S., Takahashi Y., Hachimura S. (2014). Oral administration of Lactobacillus plantarum strain AYA enhances IgA secretion and provides survival protection against influenza virus infection in mice. PLoS ONE.

[49] Kobayashi N., Saito T., Uematsu T., Kishi K., Toba M., Kohda N., Suzuki T. (2011). Oral administration of heat-killed Lactobacillus pentosus strain b240 augments protection against influenza virus infection in mice. Int. Immunopharmacol..

[50] Groeger D., Schiavi E., Grant R., Kurnik-Łucka M., Michalovich D., Williamson R., Beinke S., Kiely B., Akdis C., Hessel E. (2020). Intranasal Bifidobacterium longum protects against viral-induced lung inflammation and injury in a murine model of lethal influenza infection. EBioMedicine.

[51] Groves H.T., Higham S.L., Moffatt M.F., Cox M.J., Tregoning J.S., Bomberger J.M. (2020). Respiratory Viral Infection Alters the Gut Microbiota by Inducing Inappetence. mBio.

[52] Harding J.N., Siefker D., Vu L., You D., DeVincenzo J., Pierre J.F., Cormier S.A. (2020). Altered gut microbiota in infants is associated with respiratory syncytial virus disease severity. BMC Microbiol..

[53] Collins P.L., Melero J.A. (2011). Progress in understanding and controlling respiratory syncytial virus: Still crazy after all these years. Virus Res..

[54] Shi T., McAllister D.A., O’Brien K.L., Simoes E.A.F., Madhi S.A., Gessner B.D., Polack F.P., Balsells E., Acacio S., Aguayo C. (2017). Global, regional, and national disease burden estimates of acute lower respiratory infections due to respiratory syncytial virus in young children in 2015: A systematic review and modelling study. Lancet (Lond. Engl.).

[55] Eguchi K., Fujitani N., Nakagawa H., Miyazaki T. (2019). Prevention of respiratory syncytial virus infection with probiotic lactic acid bacterium Lactobacillus gasseri SBT2055. Sci. Rep..

[56] Chiba E., Tomosada Y., Vizoso-Pinto M.G., Salva S., Takahashi T., Tsukida K., Kitazawa H., Alvarez S., Villena J. (2013). Immunobiotic Lactobacillus rhamnosus improves resistance of infant mice against respiratory syncytial virus infection. Int. Immunopharmacol..

[57] Tomosada Y., Chiba E., Zelaya H., Takahashi T., Tsukida K., Kitazawa H., Alvarez S., Villena J. (2013). Nasally administered Lactobacillus rhamnosus strains differentially modulate respiratory antiviral immune responses and induce protection against respiratory syncytial virus infection. BMC Immunol..

[58] Ni W., Yang X., Yang D., Bao J., Li R., Xiao Y., Hou C., Wang H., Liu J., Yang D. (2020). Role of angiotensin-converting enzyme 2 (ACE2) in COVID-19. Crit. Care.

[59] Smyk W., Janik M.K., Portincasa P., Milkiewicz P., Lammert F., Krawczyk M. (2020). COVID-19: Focus on the lungs but do not forget the gastrointestinal tract. Eur. J. Clin. Investig..

[60] Jones D.L., Baluja M.Q., Graham D.W., Corbishley A., McDonald J.E., Malham S.K., Hillary L.S., Connor T.R., Gaze W.H., Moura I.B. (2020). Shedding of SARS-CoV-2 in feces and urine and its potential role in person-to-person transmission and the environment-based spread of COVID-19. Sci. Total Environ..

[61] Dang A.T., Marsland B.J. (2019). Microbes, metabolites, and the gut-lung axis. Mucosal Immunol..

[62] Thursby E., Juge N. (2017). Introduction to the human gut microbiota. Biochem. J..

[63] Fanos V., Pintus M.C., Pintus R., Marcialis M. (2020). Lung microbiota in the acute respiratory disease: From coronavirus to metabolomics. J. Pediatric Neonatal Individ. Med..

[64] Wypych T.P., Wickramasinghe L.C., Marsland B.J. (2019). The influence of the microbiome on respiratory health. Nat. Immunol..

[65] Carding S., Verbeke K., Vipond D.T., Corfe B.M., Owen L.J. (2015). Dysbiosis of the gut microbiota in disease. Microb. Ecol. Health Dis..

[66] Raftery A.L., Tsantikos E., Harris N.L., Hibbs M.L. (2020). Links between Inflammatory Bowel Disease and Chronic Obstructive Pulmonary Disease. Front. Immunol..

[67] Gu S., Chen Y., Wu Z., Chen Y., Gao H., Lv L., Guo F., Zhang X., Luo R., Huang C. (2020). Alterations of the Gut Microbiota in Patients With Coronavirus Disease 2019 or H1N1 Influenza. Clin. Infect. Dis. Off. Publ. Infect. Dis. Soc. Am..

[68] Yeoh Y.K., Zuo T. (2021). Gut microbiota composition reflects disease severity and dysfunctional immune responses in patients with COVID-19. Gut.

[69] Zuo T., Liu Q., Zhang F., Lui G.C.-Y., Tso E.Y.K., Yeoh Y.K., Chen Z., Boon S.S., Chan F.K.L., Chan P.K.S. (2021). Depicting SARS-CoV-2 faecal viral activity in association with gut microbiota composition in patients with COVID-19. Gut.

[70] Yamamoto S., Saito M., Tamura A., Prawisuda D., Mizutani T., Yotsuyanagi H. (2021). The human microbiome and COVID-19: A systematic review. PLoS ONE.

[71] Zuo T., Zhang F., Lui G.C.Y., Yeoh Y.K., Li A.Y.L., Zhan H., Wan Y., Chung A.C.K., Cheung C.P., Chen N. (2020). Alterations in Gut Microbiota of Patients With COVID-19 During Time of Hospitalization. Gastroenterology.

[72] Geva-Zatorsky N., Sefik E., Kua L., Pasman L., Tan T.G., Ortiz-Lopez A., Yanortsang T.B., Yang L., Jupp R., Mathis D. (2017). Mining the Human Gut Microbiota for Immunomodulatory Organisms. Cell.

[73] Tang L., Gu S., Gong Y., Li B., Lu H., Li Q., Zhang R., Gao X., Wu Z., Zhang J. (2020). Clinical Significance of the Correlation between Changes in the Major Intestinal Bacteria Species and COVID-19 Severity. Engineering.

[74] Fan J., Li X., Gao Y., Zhou J., Wang S., Huang B., Wu J., Cao Q., Chen Y., Wang Z. (2020). The lung tissue microbiota features of 20 deceased patients with COVID-19. J. Infect..

[75] Smythies L.E., Sellers M., Clements R.H., Mosteller-Barnum M., Meng G., Benjamin W.H., Orenstein J.M., Smith P.D. (2005). Human intestinal macrophages display profound inflammatory anergy despite avid phagocytic and bacteriocidal activity. J. Clin. Investig..

[76] Grayson M.H., Camarda L.E., Hussain S.-R.A., Zemple S.J., Hayward M., Lam V., Hunter D.A., Santoro J.L., Rohlfing M., Cheung D.S. (2018). Intestinal Microbiota Disruption Reduces Regulatory T Cells and Increases Respiratory Viral Infection Mortality Through Increased IFNγ Production. Front. Immunol..

[77] Zafar N., Aslam M., Ali A., Khatoon A., Nazir A., Tanveer Q., Bilal M., Kanwar R., Qadeer A., Sikandar M. (2020). Probiotics: Helpful for the prevention of COVID-19?. Biomed. Res. Ther..

[78] Raghuvanshi R., Grayson A.G., Schena I., Amanze O., Suwintono K., Quinn R.A. (2019). Microbial Transformations of Organically Fermented Foods. Metabolites.

[79] Hill C., Guarner F., Reid G., Gibson G.R., Merenstein D.J., Pot B., Morelli L., Canani R.B., Flint H.J., Salminen S. (2014). The International Scientific Association for Probiotics and Prebiotics consensus statement on the scope and appropriate use of the term probiotic. Nat. Rev. Gastroenterol. Hepatol..

[80] Kligler B., Cohrssen A. (2008). Probiotics. Am. Fam. Physician.

[81] Snydman D.R. (2008). The safety of probiotics. Clin. Infect. Dis. Off. Publ. Infect. Dis. Soc. Am..

[82] Gueimonde M., Ouwehand A.C., Salminen S. (2004). Safety of probiotics. Scand. J. Nutr..

[83] Sanders M.E., Akkermans L.M., Haller D., Hammerman C., Heimbach J., Hörmannsperger G., Huys G., Levy D.D., Lutgendorff F., Mack D. (2010). Safety assessment of probiotics for human use. Gut Microbes.

[84] Batista K.S., de Albuquerque J.G., de Vasconcelos M.H.A., Bezerra M.L.R., da Silva Barbalho M.B., Oliveira R.P., Aquino J.d.S. (2021). Probiotics and prebiotics: Potential prevention and therapeutic target for nutritional management of COVID-19?. Nutr. Res. Rev..

[85] Akour A. (2020). Probiotics and COVID-19: Is there any link?. Lett. Appl. Microbiol..

[86] Dhar D., Mohanty A. (2020). Gut microbiota and COVID-19- possible link and implications. Virus Res..

[87] Li Q., Cheng F., Xu Q., Su Y., Cai X., Zeng F., Zhang Y. (2021). The role of probiotics in coronavirus disease-19 infection in Wuhan: A retrospective study of 311 severe patients. Int. Immunopharmacol..

[88] Gutiérrez-Castrellón P., Gandara-Martí T., Abreu A.T., Nieto-Rufino C.D., López-Orduña E., Jiménez-Escobar I., Jiménez-Gutiérrez C., López-Velazquez G., Espadaler-Mazo J. (2021). Efficacy and safety of novel probiotic formulation in adult Covid19 outpatients: A randomized, placebo-controlled clinical trial. medRxiv.

[89] Bousquet J., Antó J., Czarlewski W., Haahtela T., Fonseca S., Iaccarino G., Blain H., Vidal A., Sheikh A., Akdis C. (2020). Cabbage and fermented vegetables: From death rate heterogeneity in countries to candidates for mitigation strategies of severe COVID-19. Allergy.

[90] Hemarajata P., Versalovic J. (2013). Effects of probiotics on gut microbiota: Mechanisms of intestinal immunomodulation and neuromodulation. Therap. Adv. Gastroenterol..

[91] Cho I., Blaser M.J. (2012). The human microbiome: At the interface of health and disease. Nat. Rev. Genet..

[92] Eetemadi A., Rai N., Pereira B.M.P., Kim M., Schmitz H., Tagkopoulos I. (2020). The Computational Diet: A Review of Computational Methods Across Diet, Microbiome, and Health. Front. Microbiol..

[93] Satokari R. (2019). Modulation of Gut Microbiota for Health by Current and Next-Generation Probiotics. Nutrients.

[94] Singh R., Chang H.-W., Yan D., Lee K., Ucmak D., Wong K., Abrouk M., Farahnik B., Nakamura M., Zhu T. (2017). Influence of diet on the gut microbiome and implications for human health. J. Transl. Med..

[95] Walker A., Pfitzner B., Neschen S., Kahle M., Harir M., Lucio M., Moritz F., Tziotis D., Witting M., Rothballer M. (2014). Distinct signatures of host-microbial meta-metabolome and gut microbiome in two C57BL/6 strains under high-fat diet. ISME J..

[96] Zhang X., Deeke S.A., Ning Z., Starr A.E., Butcher J., Li J., Mayne J., Cheng K., Liao B., Li L. (2018). Metaproteomics reveals associations between microbiome and intestinal extracellular vesicle proteins in pediatric inflammatory bowel disease. Nat. Commun..

[97] Lavelle A., Sokol H. (2018). Gut microbiota: Beyond metagenomics, metatranscriptomics illuminates microbiome functionality in IBD. Nature reviews. Gastroenterol. Hepatol..

[98] Wang W.L., Xu S.Y., Ren Z.G., Tao L., Jiang J.W., Zheng S.S. (2015). Application of metagenomics in the human gut microbiome. World J. Gastroenterol..

[99] Shim J.S., Oh K., Kim H.C. (2014). Dietary assessment methods in epidemiologic studies. Epidemiol. Health.

[100] Peterson J., Garges S., Giovanni M., McInnes P., Wang L., Schloss J.A., Bonazzi V., McEwen J.E., Wetterstrand K.A., Deal C. (2009). The NIH Human Microbiome Project. Genome Res..

[101] Cheng L., Qi C., Zhuang H., Fu T., Zhang X. (2019). gutMDisorder: A comprehensive database for dysbiosis of the gut microbiota in disorders and interventions. Nucleic Acids Res..

[102] Li L., Jing Q., Yan S., Liu X., Sun Y., Zhu D., Wang D., Hao C., Xue D. (2021). Amadis: A Comprehensive Database for Association Between Microbiota and Disease. Front. Physiol..

[103] Hiseni P., Rudi K., Wilson R.C., Hegge F.T., Snipen L. (2021). HumGut: A comprehensive human gut prokaryotic genomes collection filtered by metagenome data. Microbiome.

[104] Chen Y.-A., Park J., Natsume-Kitatani Y., Kawashima H., Mohsen A., Hosomi K., Tanisawa K., Ohno H., Konishi K., Murakami H. (2020). MANTA, an integrative database and analysis platform that relates microbiome and phenotypic data. PLoS ONE.

[105] King C.H., Desai H., Sylvetsky A.C., LoTempio J., Ayanyan S., Carrie J., Crandall K.A., Fochtman B.C., Gasparyan L., Gulzar N. (2019). Baseline human gut microbiota profile in healthy people and standard reporting template. PLoS ONE.

[106] Friede A., Reid J.A., Ory H.W. (1993). CDC WONDER: A comprehensive on-line public health information system of the Centers for Disease Control and Prevention. Am. J. Public Health.

[107] Imhann F., Van der Velde K.J., Barbieri R., Alberts R., Voskuil M.D., Vich Vila A., Collij V., Spekhorst L.M., Van der Sloot K.W.J., Peters V. (2019). The 1000IBD project: Multi-omics data of 1000 inflammatory bowel disease patients; data release 1. BMC Gastroenterol..

[108] Freifeld C.C., Mandl K.D., Reis B.Y., Brownstein J.S. (2008). HealthMap: Global Infectious Disease Monitoring through Automated Classification and Visualization of Internet Media Reports. J. Am. Med. Inform. Assoc..

[109] Kim S.K., Guevarra R.B., Kim Y.T., Kwon J., Kim H., Cho J.H., Kim H.B., Lee J.H. (2019). Role of Probiotics in Human Gut Microbiome-Associated Diseases. J. Microbiol. Biotechnol..

[110] Ursell L.K., Metcalf J.L., Parfrey L.W., Knight R. (2012). Defining the human microbiome. Nutr. Rev..

[111] Shreiner A.B., Kao J.Y., Young V.B. (2015). The gut microbiome in health and in disease. Curr. Opin. Gastroenterol..

[112] Segata N., Waldron L., Ballarini A., Narasimhan V., Jousson O., Huttenhower C. (2012). Metagenomic microbial community profiling using unique clade-specific marker genes. Nat. Methods.

[113] Meyer F., Paarmann D., D’Souza M., Olson R., Glass E.M., Kubal M., Paczian T., Rodriguez A., Stevens R., Wilke A. (2008). The metagenomics RAST server—A public resource for the automatic phylogenetic and functional analysis of metagenomes. BMC Bioinform..

[114] Li D., Liu C.-M., Luo R., Sadakane K., Lam T.-W. (2015). MEGAHIT: An ultra-fast single-node solution for large and complex metagenomics assembly via succinct de Bruijn graph. Bioinformatics.

[115] Franzosa E.A., McIver L.J., Rahnavard G., Thompson L.R., Schirmer M., Weingart G., Lipson K.S., Knight R., Caporaso J.G., Segata N. (2018). Species-level functional profiling of metagenomes and metatranscriptomes. Nat. Methods.

[116] Kuczynski J., Stombaugh J., Walters W.A., González A., Caporaso J.G., Knight R. (2011). Using QIIME to analyze 16S rRNA gene sequences from microbial communities. Curr. Protoc. Bioinform..

[117] Schloss P.D., Westcott S.L., Ryabin T., Hall J.R., Hartmann M., Hollister E.B., Lesniewski R.A., Oakley B.B., Parks D.H., Robinson C.J. (2009). Introducing mothur: Open-source, platform-independent, community-supported software for describing and comparing microbial communities. Appl. Environ. Microbiol..

[118] Bankevich A., Nurk S., Antipov D., Gurevich A.A., Dvorkin M., Kulikov A.S., Lesin V.M., Nikolenko S.I., Pham S., Prjibelski A.D. (2012). SPAdes: A new genome assembly algorithm and its applications to single-cell sequencing. J. Comput. Biol. J. Comput. Mol. Cell Biol..

[119] Luo R., Liu B., Xie Y., Li Z., Huang W., Yuan J., He G., Chen Y., Pan Q., Liu Y. (2015). Erratum: SOAPdenovo2: An empirically improved memory-efficient short-read de novo assembler. GigaScience.

[120] Westreich S.T., Treiber M.L., Mills D.A., Korf I., Lemay D.G. (2018). SAMSA2: A standalone metatranscriptome analysis pipeline. BMC Bioinform..

[121] Milanese A., Mende D.R., Paoli L., Salazar G., Ruscheweyh H.-J., Cuenca M., Hingamp P., Alves R., Costea P.I., Coelho L.P. (2019). Microbial abundance, activity and population genomic profiling with mOTUs2. Nat. Commun..

[122] Zhang H., Ai J.W., Yang W., Zhou X., He F., Xie S., Zeng W., Li Y., Yu Y., Gou X. (2021). Metatranscriptomic Characterization of Coronavirus Disease 2019 Identified a Host Transcriptional Classifier Associated With Immune Signaling. Clin. Infect. Dis..

[123] Shen Z., Xiao Y., Kang L., Ma W., Shi L., Zhang L., Zhou Z., Yang J., Zhong J., Yang D. (2020). Genomic Diversity of Severe Acute Respiratory Syndrome-Coronavirus 2 in Patients With Coronavirus Disease 2019. Clin. Infect. Dis. Off. Publ. Infect. Dis. Soc. Am..

[124] Mohs R.C., Greig N.H. (2017). Drug discovery and development: Role of basic biological research. Alzheimer’s Dement. (N. Y.).

[125] Dibyajyoti S., Bin E.T., Swati P.P. (2013). Bioinformatics:The effects on the cost of drug discovery. Galle Med. J..

[126] Osakwe O. (2016). The Significance of Discovery Screening and Structure Optimization Studies. Social Aspects of Drug Discovery, Development and Commercialization.

[127] Clark D.E. (2006). What has computer-aided molecular design ever done for drug discovery?. Expert Opin. Drug Discov..

[128] Kirchmair J., Distinto S., Liedl K.R., Markt P., Rollinger J.M., Schuster D., Spitzer G.M., Wolber G. (2011). Development of anti-viral agents using molecular modeling and virtual screening techniques. Infect. Disord. Drug Targets.

[129] Kumar V., Chandra S., Siddiqi M.I. (2014). Recent advances in the development of antiviral agents using computer-aided structure based approaches. Curr. Pharm. Des..

[130] Selvaraj G.F., Piramanayagam S., Devadasan V., Hassan S., Krishnasamy K., Srinivasan S. (2020). Computational analysis of drug like candidates against Neuraminidase of Human Influenza A virus subtypes. Inform. Med. Unlocked.

[131] Mallipeddi P.L., Kumar G., White S.W., Webb T.R. (2014). Recent advances in computer-aided drug design as applied to anti-influenza drug discovery. Curr. Top. Med. Chem..

[132] Mottin M., Borba J., Melo-Filho C., Neves B., Muratov E., Torres P., Braga R., Perryman A., Ekins S., Andrade C. (2018). Computational Drug Discovery for the Zika Virus. Braz. J. Pharm. Sci..

[133] Tahir ul Qamar M., Maryam A., Muneer I., Xing F., Ashfaq U.A., Khan F.A., Anwar F., Geesi M.H., Khalid R.R., Rauf S.A. (2019). Computational screening of medicinal plant phytochemicals to discover potent pan-serotype inhibitors against dengue virus. Sci. Rep..

[134] Alizon S., Cazals F., Guindon S., Lemaitre C., Mary-Huard T., Niarakis A., Salson M., Scornavacca C., Touzet H. (2021). SARS-CoV-2 Through the Lens of Computational Biology: How Bioinformatics Is Playing a Key Role in the Study of the Virus and Its Origins.

[135] Mhatre S., Naik S., Patravale V. (2021). A molecular docking study of EGCG and theaflavin digallate with the druggable targets of SARS-CoV-2. Comput. Biol. Med..

[136] Kim S., Chen J., Cheng T., Gindulyte A., He J., He S., Li Q., Shoemaker B., Thiessen P., Yu B. (2018). PubChem 2019 update: Improved access to chemical data. Nucleic Acids Res..

[137] Irwin J.J., Shoichet B.K. (2005). ZINC—a free database of commercially available compounds for virtual screening. J. Chem. Inf. Modeling.

[138] Morris G.M., Goodsell D.S., Halliday R.S., Huey R., Hart W.E., Belew R.K., Olson A.J. (1998). Automated docking using a Lamarckian genetic algorithm and an empirical binding free energy function. J. Comput. Chem..

[139] Trott O., Olson A.J. (2010). AutoDock Vina: Improving the speed and accuracy of docking with a new scoring function, efficient optimization, and multithreading. J. Comput. Chem..

[140] Rarey M., Kramer B., Lengauer T., Klebe G. (1996). A fast flexible docking method using an incremental construction algorithm. J. Mol. Biol..

[141] Friesner R.A., Banks J.L., Murphy R.B., Halgren T.A., Klicic J.J., Mainz D.T., Repasky M.P., Knoll E.H., Shelley M., Perry J.K. (2004). Glide: A new approach for rapid, accurate docking and scoring. 1. Method and assessment of docking accuracy. J. Med. Chem..

[142] Vardhan S., Sahoo S.K. (2020). In silico ADMET and molecular docking study on searching potential inhibitors from limonoids and triterpenoids for COVID-19. Comput. Biol. Med..

[143] Suručić R., Tubić B., Stojiljković M.P., Djuric D.M., Travar M., Grabež M., Šavikin K., Škrbić R. (2021). Computational study of pomegranate peel extract polyphenols as potential inhibitors of SARS-CoV-2 virus internalization. Mol. Cell. Biochem..

[144] Yu R., Chen L., Lan R., Shen R., Li P. (2020). Computational screening of antagonists against the SARS-CoV-2 (COVID-19) coronavirus by molecular docking. Int. J. Antimicrob. Agents.

[145] Anwar F., Altayb H.N., Al-Abbasi F.A., Al-Malki A.L., Kamal M.A., Kumar V. (2021). Antiviral effects of probiotic metabolites on COVID-19. J. Biomol. Struct. Dyn..

[146] Balmeh N., Mahmoudi S., Fard N.A. (2021). Manipulated bio antimicrobial peptides from probiotic bacteria as proposed drugs for COVID-19 disease. Inform. Med. Unlocked.

[147] Rather I., Choi S.B., Kamli M., Hakeem K., Sabir J., Park Y.-H., Hor Y.-Y., Leitão J. (2021). Potential Adjuvant Therapeutic Effect of Lactobacillus plantarum Probio-88 Postbiotics against SARS-CoV-2. Vaccines.

[148] Pires D.E., Blundell T.L., Ascher D.B. (2015). pkCSM: Predicting Small-Molecule Pharmacokinetic and Toxicity Properties Using Graph-Based Signatures. J. Med. Chem..

[149] Husain A., Ahmad A., Khan S.A., Asif M., Bhutani R., Al-Abbasi F.A. (2016). Synthesis, molecular properties, toxicity and biological evaluation of some new substituted imidazolidine derivatives in search of potent anti-inflammatory agents. Saudi Pharm. J..

[150] Ravindranath P.A., Forli S., Goodsell D.S., Olson A.J., Sanner M.F. (2015). AutoDockFR: Advances in Protein-Ligand Docking with Explicitly Specified Binding Site Flexibility. PLoS Comput. Biol..

[151] Sonawane P., Patel K., Vishwakarma R., Singh S., Khan B. (2013). In Silico mutagenesis and Docking studies of active site residues suggest altered substrate specificity and possible physiological role of Cinnamoyl CoA Reductase 1 (Ll-CCRH1). Bioinformation.

[152] Rodrigues C.H., Pires D.E., Ascher D.B. (2018). DynaMut: Predicting the impact of mutations on protein conformation, flexibility and stability. Nucleic Acids Res..

[153] Jo S., Kim T., Iyer V.G., Im W. (2008). CHARMM-GUI: A web-based graphical user interface for CHARMM. J. Comput. Chem..

[154] Humphrey W., Dalke A., Schulten K. (1996). VMD: Visual molecular dynamics. J. Mol. Graph..

[155] Volkamer A., Kuhn D., Rippmann F., Rarey M. (2012). DoGSiteScorer: A web-server for automatic binding site prediction, analysis, and druggability assessment. Bioinformatics.

[156] Pawar S., Rohane S. (2021). Review on Discovery Studio: An important Tool for Molecular Docking. Asian J. Res. Chem..

[157] Biasini M., Bienert S., Waterhouse A., Arnold K., Studer G., Schmidt T., Kiefer F., Gallo Cassarino T., Bertoni M., Bordoli L. (2014). SWISS-MODEL: Modelling protein tertiary and quaternary structure using evolutionary information. Nucleic Acids Res..

[158] Williams C., Headd J., Moriarty N., Prisant M., Videau L., Deis L., Verma V., Keedy D., Hintze B., Chen V. (2017). MolProbity: More and better reference data for improved all-atom structure validation. Protein Sci..

[159] Laskowski R.A., Rullmannn J.A., MacArthur M.W., Kaptein R., Thornton J.M. (1996). AQUA and PROCHECK-NMR: Programs for checking the quality of protein structures solved by NMR. J. Biomol. NMR.

[160] Eisenberg D., Lüthy R., Bowie J.U. (1997). VERIFY3D: Assessment of protein models with three-dimensional profiles. Methods Enzymol..

[161] Hooft R.W., Vriend G., Sander C., Abola E.E. (1996). Errors in protein structures. Nature.

[162] Salentin S., Schreiber S., Haupt V.J., Adasme M.F., Schroeder M. (2015). PLIP: Fully automated protein-ligand interaction profiler. Nucleic Acids Res..

[163] Elfiky A.A. (2020). Ribavirin, Remdesivir, Sofosbuvir, Galidesivir, and Tenofovir against SARS-CoV-2 RNA dependent RNA polymerase (RdRp): A molecular docking study. Life Sci..

[164] Eswar N., Eramian D., Webb B., Shen M.-Y., Sali A., Kobe B., Guss M., Huber T. (2008). Protein Structure Modeling with MODELLER. Structural Proteomics: High-Throughput Methods.

[165] Yang H., Lou C., Sun L., Li J., Cai Y., Wang Z., Li W., Liu G., Tang Y. (2018). admetSAR 2.0: Web-service for prediction and optimization of chemical ADMET properties. Bioinformatics.

[166] Aftab O., Ghouri M., Masood M., Haider Z., Khan Z., Ahmad A., Munawar N. (2020). Analysis of SARS-CoV-2 RNA-dependent RNA polymerase as a potential therapeutic drug target using a computational approach. J. Transl. Med..

[167] Pettersen E.F., Goddard T.D., Huang C.C., Couch G.S., Greenblatt D.M., Meng E.C., Ferrin T.E. (2004). UCSF Chimera—A visualization system for exploratory research and analysis. J. Comput. Chem..

[168] Morris G.M., Huey R., Lindstrom W., Sanner M.F., Belew R.K., Goodsell D.S., Olson A.J. (2009). AutoDock4 and AutoDockTools4: Automated docking with selective receptor flexibility. J. Comput. Chem..

[169] Laskowski R.A., Swindells M.B. (2011). LigPlot+: Multiple ligand-protein interaction diagrams for drug discovery. J. Chem. Inf. Modeling.

[170] Abraham M.J., Murtola T., Schulz R., Páll S., Smith J.C., Hess B., Lindahl E. (2015). GROMACS: High performance molecular simulations through multi-level parallelism from laptops to supercomputers. SoftwareX.

[171] Schmid N., Eichenberger A., Choutko A., Riniker S., Winger M., Mark A., van Gunsteren W. (2011). Definition and testing of the GROMOS force-field versions 54A7 and 54B7. Eur. Biophys. J. EBJ.

[172] Behera S.K., Vhora N., Contractor D., Shard A., Kumar D., Kalia K., Jain A. (2021). Computational drug repurposing study elucidating simultaneous inhibition of entry and replication of novel corona virus by Grazoprevir. Sci. Rep..

[173] van Zundert G.C.P., Rodrigues J., Trellet M., Schmitz C., Kastritis P.L., Karaca E., Melquiond A.S.J., van Dijk M., de Vries S.J., Bonvin A. (2016). The HADDOCK2.2 Web Server: User-Friendly Integrative Modeling of Biomolecular Complexes. J. Mol. Biol..

[174] Scialò F., Daniele A., Amato F., Pastore L., Matera M.G., Cazzola M., Castaldo G., Bianco A. (2020). ACE2: The Major Cell Entry Receptor for SARS-CoV-2. Lung.

[175] Baughn L.B., Sharma N., Elhaik E., Sekulic A., Bryce A.H., Fonseca R. (2020). Targeting TMPRSS2 in SARS-CoV-2 Infection. Mayo Clin. Proc..

[176] Hoffmann M., Kleine-Weber H., Schroeder S., Krüger N., Herrler T., Erichsen S., Schiergens T.S., Herrler G., Wu N.H., Nitsche A. (2020). SARS-CoV-2 Cell Entry Depends on ACE2 and TMPRSS2 and Is Blocked by a Clinically Proven Protease Inhibitor. Cell.

[177] Huang Y., Yang C., Xu X.-f., Xu W., Liu S.-w. (2020). Structural and functional properties of SARS-CoV-2 spike protein: Potential antivirus drug development for COVID-19. Acta Pharmacol. Sin..

[178] Osipiuk J., Azizi S.-A., Dvorkin S., Endres M., Jedrzejczak R., Jones K.A., Kang S., Kathayat R.S., Kim Y., Lisnyak V.G. (2021). Structure of papain-like protease from SARS-CoV-2 and its complexes with non-covalent inhibitors. Nat. Commun..

[179] Tahir Ul Qamar M., Alqahtani S.M., Alamri M.A., Chen L.L. (2020). Structural basis of SARS-CoV-2 3CL(pro) and anti-COVID-19 drug discovery from medicinal plants. J. Pharm. Anal..

[180] Zhu W., Chen C.Z., Gorshkov K., Xu M., Lo D.C., Zheng W. (2020). RNA-Dependent RNA Polymerase as a Target for COVID-19 Drug Discovery. SLAS DISCOVERY Adv. Sci. Drug Discov..

[181] Weber R., McCullagh M. (2021). Role of ATP in the RNA Translocation Mechanism of SARS-CoV-2 NSP13 Helicase. J. Phys. Chem. B.

[182] Habtemariam S., Nabavi S.F., Banach M., Berindan-Neagoe I., Sarkar K., Sil P.C., Nabavi S.M. (2020). Should We Try SARS-CoV-2 Helicase Inhibitors for COVID-19 Therapy?. Arch. Med. Res..

[183] Spratt A., Gallazzi F., Quinn T., Lorson C., Sönnerborg A., Singh K. (2021). Coronavirus helicases: Attractive and unique targets of antiviral drug-development and therapeutic patents. Expert Opin. Ther. Pat..

[184] Ekblad B., Kristiansen P.E. (2019). NMR structures and mutational analysis of the two peptides constituting the bacteriocin plantaricin S. Sci. Rep..

[185] Ekblad B., Kyriakou P.K., Oppegård C., Nissen-Meyer J., Kaznessis Y.N., Kristiansen P.E. (2016). Structure–Function Analysis of the Two-Peptide Bacteriocin Plantaricin EF. Biochemistry.

[186] Fimland N., Rogne P., Fimland G., Nissen-Meyer J., Kristiansen P.E. (2008). Three-dimensional structure of the two peptides that constitute the two-peptide bacteriocin plantaricin EF. Biochim. Biophys. Acta.

[187] Selegård R., Musa A., Nyström P., Aili D., Bengtsson T., Khalaf H. (2019). Plantaricins markedly enhance the effects of traditional antibiotics against Staphylococcus epidermidis. Future Microbiol..

[188] (2021). AlphaFold Colab. https://colab.research.google.com/github/deepmind/alphafold/blob/main/notebooks/AlphaFold.ipynb.

[189] Jumper J., Evans R., Pritzel A., Green T., Figurnov M., Ronneberger O., Tunyasuvunakool K., Bates R., Žídek A., Potapenko A. (2021). Highly accurate protein structure prediction with AlphaFold. Nature.

[190] Yuan S., Chan H.C.S., Hu Z. (2017). Using PyMOL as a platform for computational drug design. WIREs Comput. Mol. Sci..

[191] Webb B., Sali A. (2016). Comparative Protein Structure Modeling Using MODELLER. Curr. Protoc. Bioinform..

